# Phytocannabinoids: Pharmacological effects, biomedical applications, and worldwide prospection

**DOI:** 10.1016/j.jtcme.2023.08.006

**Published:** 2023-08-26

**Authors:** Ana L.G. de Brito Siqueira, Pedro V.V. Cremasco, Juliana O. Bahú, Aline Pioli da Silva, Lucas R. Melo de Andrade, Paula G.A. González, Sara Crivellin, Viktor O. Cárdenas Concha, Karolline Krambeck, Leandro Lodi, Patrícia Severino, Eliana B. Souto

**Affiliations:** a Institute of Science and Technology, Federal University of Alfenas (UNIFAL), Poços de Caldas, 37715-400, Minas Gerais, Brazil; b National Institute of Science and Technology in Biofabrication (INCT-BIOFABRIS), School of Chemical Engineering, University of Campinas, Albert Einstein Ave., Cidade Universitária Zeferino Vaz, Campinas, 13083-852, SP, Brazil; c Institute of Environmental, Chemical and Pharmaceutical Science, School of Chemical Engineering, Federal University of São Paulo (UNIFESP), São Nicolau St., Jd. Pitangueiras, Diadema, 09913-030, SP, Brazil; d Laboratory of Pharmaceutical Technology, Faculty of Pharmaceutical Sciences, Food and Nutrition, Federal University of Mato Grosso do Sul, Campo Grande, 79070-900, MS, Brazil; e Laboratory of Pharmaceutical Technology, Department of Drug Sciences, Faculty of Pharmacy, University of Porto, 4050-313, Porto, Portugal; f UCIBIO – Applied Molecular Biosciences Unit, MEDTECH, Faculty of Pharmacy, University of Porto, 4050-313, Porto, Portugal; g Associate Laboratory i4HB - Institute for Health and Bioeconomy, Faculty of Pharmacy, University of Porto, 4050-313, Porto, Portugal; h Laboratory of Nanotechnology and Nanomedicine (LNMed), Institute of Technology and Research (ITP), Murilo Dantas Ave., 300, Aracaju, 49010-390, Sergipe, Brazil; i Industrial Biotechnology Program, University of Tiradentes (UNIT), Murilo Dantas Ave., 300, Aracaju, 49010-390, Sergipe, Brazil

**Keywords:** Cannabidiol, *Cannabis Sativa* L., Commercial products, Market, Trans-Delta-9-tetrahydrocannabinol

## Abstract

Scientific evidence exists about the association between neurological diseases (i.e., Parkinson's disease, Alzheimer's disease, amyotrophic lateral sclerosis (ALS), multiple sclerosis, depression, and memory loss) and oxidative damage. The increasing worldwide incidence of such diseases is attracting the attention of researchers to find palliative medications to reduce the symptoms and promote quality of life, in particular, in developing countries, e.g., South America and Africa. Among potential alternatives, extracts of *Cannabis Sativa* L. are suitable for people who have neurological disorders, spasticity, and pain, nausea, resulting from diseases such as cancer and arthritis. In this review, we discuss the latest developments in the use of Cannabis, its subtypes and constituents, extraction methods, and relevant pharmacological effects. Biomedical applications, marketed products, and prospects for the worldwide use of *Cannabis Sativa* L. extracts are also discussed, providing the bibliometric maps of scientific literature published in representative countries from South America (i.e., Brazil) and Africa (i.e., South Africa). A lack of evidence on the effectiveness and safety of Cannabis, besides the concerns about addiction and other adverse events, has led many countries to act with caution before changing Cannabis-related regulations. Recent findings are expected to increase the social acceptance of Cannabis, while new technologies seem to boost the global cannabis market because the benefits of (−)-trans-delta-9-tetrahydrocannabinol (Δ9-THC) and cannabidiol (CBD) use have been proven in several studies in addition to the potential to general new employment.

## Graphical abstract

**Figure:**
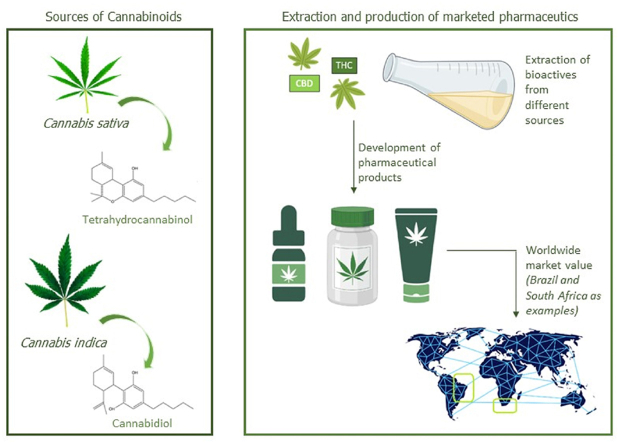

## 1 Introduction

The literal definition of Marihuana/Cannabis can be found in the United States Code (USC): “All parts of the plant *Cannabis Sativa* L., whether growing or not; the seeds thereof; the resin extracted from any part of such plant; and every compound, manufacture, salt, derivative, mixture, or preparation of such plant, its seeds or resin. Such term does not include the mature stalks of the plant, fiber produced from such stalks, oil or cake made from the seeds of such plant, any other compound, manufacture, salt, derivative, mixture, or preparation of such mature stalks (except the resin extracted therefrom), fiber, oil, or cake, or the sterilized seed of this plant which is incapable of germination”^1^

Early, the Cannabis plants had been used as food and textile fibers, but currently, it is cultivated and distributed worldwide. Cannabis is a genus of plants in the *Cannabaceae* family, that is a semi-herb, annual, and dioecious flowering herb, having three subspecies (Table 1): *sativa*, *indica*, and *ruderalis* (rare).^2^^,^^3^

**Table 1:** Main differences between the *Cannabis* subspecies (adapted from .^4^^,^^5^

Indica	Ruderalis	Sativa
		
*Indica* plants have a short and bushy appearance, with branches that are short and heavy with thick, dense buds. These buds mature early, typically in early September in the Northern Hemisphere. The color of *Indica* buds can range from dark green to purple, with cooler temperatures causing more intense coloration.	*Ruderalis* is rarely cultivated for the production of pharmaceutics (THC <0.3%). This subspecies is used to create *Sativa* or *Indica* hybrids with select desired traits. Its seeds are utilized in the creation of various products, including food, nutritional supplements, cosmetics, and medicines, while its stem and fibers are used by the industry to produce paper, fabrics, ropes, bioplastic compounds, biofuels, and construction materials.	*Sativa* leaves are smaller and thinner in comparison to *Indica* leaves, which have wide fingers and are a deep green color, often with a purple tint. As the *Indica* plant matures, its leaves turn a dark purple hue. Its plants tend to be taller and produce fewer flowers.

Cannabis is derived from dicotyledonous (flowering plants that have two leaves when they germinate), herbaceous (non-woody plants whose aerial parts die after fruiting), dioecious (male plants differ from female plants), apetalous (flowers with no corolla). *Cannabis sativa* L. and its variants biosynthesize the terpene phenolic cannabinoids or phytocannabinoids exclusively, that are accumulated in the plant's glandular trichomes.^6^, ^7^, ^8^ The female plants and flowers are small and resistant, germinating axially and terminally, with their ovaries found in bracts and their pollination being carried out by the wind. The male plants and flowers are small, with five greenish sepals and five stamens that when opened release pollen.^9^

Cannabis composition is complex, it has more than 500 chemical compounds identified until now, among them, 120 are classified as phytocannabinoids, and other phytochemicals covering non-cannabinoid terpenoids and phenolics, fatty acids, organic acids, amino acids, fatty acids, ketones, esters/lactones, alcohols, alkaloids, vitamins, and polysaccharides.^10^, ^11^, ^12^

The policy for using cannabis as a medicine has changed dramatically in recent years. Still, a lack of evidence on the effectiveness and safety of cannabis, as well as concerns about addiction and other adverse events, has led many countries to exercise caution before changing cannabis regulations. Herbal cannabis is banned in most countries in Europe, and cannabinoid-based drugs are legal in many of them. Only three cannabinoid medicines are currently available for sale in different countries.^13^

### 1.1 Cannabinoids

The term “cannabinoids” refers not only to chemicals with a typical C21 terpene phenolic backbone isolated from *C. sativa* L. but also to their derivatives, with the term phytocannabinoids being used for plant extract.^14^

Cannabinoids can be classified into three distinct classes:

1. Phytocannabinoids: naturally found in plants;
2. Endocannabinoids naturally produced within the body;
3. Synthetic cannabinoids: cannabinomimetic compounds resulting from chemical synthesis.^7^^,^^15^

### 1.2 Phytocannabinoids

The term phytocannabinoid defines meroterpenoids with a resorcinol core typically decorated with a para-positioned isoprenyl, alkyl, or aralkyl side chain.^7^^,^^16^

Alkyl side chains usually contain an odd carbon atoms number, with orcinoids containing one carbon, varinoids three, and olivetoids five. Cannabinoids that have an even number of carbon atoms in their side chain do exist, but they are rare. The term cannabinoid usually refers to a molecule with a characteristic chemical structure; however, the term can also refer to the pharmacological ligands of human endocannabinoid receptors.^17^^,^^18^

**Fig. 1:**
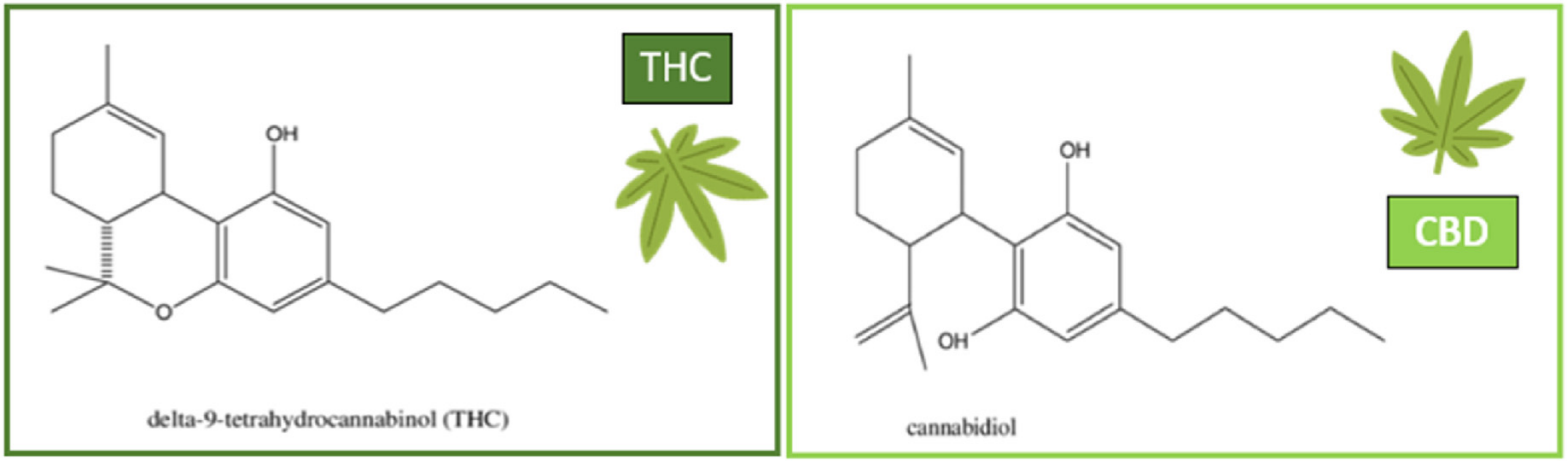
Molecules of Cannabinoids (adapted from Mechoulam (2019)^7^; Russo and Guy, (2006) ^21^).

The most studied extracts from phytocannabinoids are (−)-trans-delta-9-tetrahydrocannabinol (Δ9-THC) and cannabidiol (CBD) (Fig. 1).^19^^,^^20^

Some examples of phytocannabinoids' use are the treatment of malignant brain tumors, Parkinson's disease (PD), Alzheimer's disease (AD), multiple sclerosis (MS), neuropathic pain, childhood epilepsy, Lennox-Gastaut, and Dravet syndromes.^22^, ^23^, ^24^ In this review, we present data from animal/human studies on the current clinical/neurological uses of CBD alone or in combination with Δ9-THC, highlighting its use in various clinical neuroprotective, anti-inflammatory, and immunomodulatory benefits when used in the environment.^25^, ^26^, ^27^

## 2 Extraction processes

There are several processes applied for the extraction of cannabinoids, terpenes, and fatty acids from cannabis, including distillation, Soxhlet extraction, ultrasound, and microwave, among others.^28^ However, some drawbacks are related to these processes, such as yielding dependent on the extracting solvent used, thermal degradation accompanied by decarboxylation, need for “downstream” separation and purification steps. Together, these factors reduce the process efficiency, and final product purity, and increase costs as other process steps are required for the products to achieve the minimum specification. A traditional process for cannabis extraction is supercritical carbon dioxide (CO_2_) or water since these solvents are not toxic. The supercritical process also has some advantages concerning operation economy, environmental concerns, as well as purification steps, are no need to obtain products with high purity.^29^^,^^30^ Some supercritical extraction methods based on green solvents are summarized in Table 2.

**Table 2:** Cannabis supercritical extraction methods.

Conditions	Results	Reference
Material: *Cannabis sativa* seeds; Solvent: Water; Pressure: 105 bar; Temperature: 80-200 °C	Low levels of Δ9-THC and CBN were achieved, yielding a nutraceutical-grade cannabinoid	^31^
Material: Cannabis bars; Solvent: Carbon dioxide (CO_2_); Pressure: 180 bar; Temperature: 40 °C	Yielding: ≈14% wt (CBD), ≈23% wt (THC) – in the extract	^32^
Material: Cannabis from different regions, Solvent: CO_2_; Co-Solvent: Ethanol (2 % wt); Pressure: 330 bar; Temperature: 40 °C, Time: 2 h	Yielding: ≈2% wt (CBD), ≈27% wt (THC)	^33^
Material: *Cannabis Sativa* L.; Solvent: CO_2_; Pressure: 380 bar; Temperature: 60–80 °C	After the purification process, it was possible to obtain a CBD extract with 80% purity	^34^
Material: Hemp plant; Solvent: CO_2_; Co-Solvent: Ethanol; Pressure: 200 bar; Temperature: 40 °C	CBD extract (≈449 mg/g)	^35^
Material: Cannabis bud material; Solvent: CO_2_; Pressure: 320 bar; Temperature: 60 °C; Time: 600 min; Flow Rate: 150 g/min	Extraction: ≈7% wt, with the highest recoveries for CBD and THC (>98%)	^36^
Material: *Cannabis sativa* L.; Solvent: CO_2_; Co-Solvent: Ethanol (0–5% wt); Pressure: 150–330 bar; Temperature: 60 °C; Time: 600 min; Flow Rate: 150 g/min	THC yields ≈ 37% Purity ≈ 90%	^37^
Material: *Cannabis sativa* L.; Solvent: CO_2_; Pressure: 170–340 bar; Temperature: 55 °C; Flow Rate: 200 g/min	THCA and Δ9-THC Process extraction efficiency ≈92%	^38^
Material: Cannabis flowers; Solvent: CO_2_; Pressure: 250 bar; Temperature: 45 °C; Time: 180 min	Concentration: ≈2.4% wt (CBDA), ≈0.05% wt (THCA)	^39^

Several extraction processes are being studied and among them, we can highlight as main ones: the use of solvents in the liquid phase, distillation, Soxhlet extraction, ultrasound, microwave, and supercritical fluids, the latter with the possibility of using co-solvents. The following variables are relevant:

- Maximizing the yield of the product obtained after extraction. In this case, the processes which are considered less selective (solvents in the liquid phase, distillation, Soxhlet extraction, ultrasound, microwave) produce a good yield in the extract, but produce an extract with low purity, relative to the cannabinoids. In this case, is possible to estimate a low cost in the extraction process, but a high cost in the “downstream” purification process which can add many steps with a high cost of process implementation.
- Degree of purity of the compounds of interest. In this case, the supercritical extraction processes, considered of good selectivity concerning the compounds of interest, can extract with a higher degree of purity due to the increased selectivity of the solvent that is characteristic of this type of process. Having, once again, the possibility of obtaining a product with a better degree of purity and lower production cost.^40^
- Minimizing the possibility of product contamination with process feedstock to decrease “downstream” purification steps. For this analysis, it is possible to highlight extractions with water in the liquid phase or distillation, ultrasound, microwave, and supercritical CO_2_ extraction (SC–CO_2_). Again, the supercritical SC-CO_2_ process seems to have advantages because it is a solvent considered to be non-contaminant and the separation process is simple, being only necessary to decrease the pressure with the passage of this solvent to the gaseous state leaving the sample free of any residue of this solvent.^41^^,^^42^
- Scalability for industrial processes. For this analysis, it is possible to determine the possibility of all processes pointed out to be possible to scale up. As discussed above what differentiates each process again may be the costs considering the extraction and especially the downstream purification steps.

Finally, supercritical extraction processes, especially using CO_2_ (SC–CO_2_), seem to have the greatest advantages, which is perhaps why they have been widely studied for cannabinoid extraction.

## 3 Pharmacological effects

*Cannabis sativa* plant has a long history related to therapeutic and pharmacological use by humans. Despite having more than 400 chemical compounds, its two main constituents are cannabidiol (CBD) and tetrahydrocannabinol (THC) responsible for the main pharmacological actions.^43^

Cannabidiol (CBD) is a non-psychotomimetic phytocannabinoid being the second most present chemical compound in cannabis. This compound was initially isolated in 1940, described as non-psychomimetic, has a relatively low bioavailability in intravenous (IV) administrations, and can reach the blood-brain barrier (BBB) in addition to having a tolerable and acceptable safety profile for use in the treatment of diseases. related to the central nervous system (CNS). CBD shows some pharmacological activities already reported in the literature, for example, anxiolytic, anticonvulsant, anti-nausea, anti-inflammatory, and anti-rheumatoid activity.^44^, ^45^, ^46^, ^47^, ^48^, ^49^

In addition to several activities, CBD has attracted the attention of researchers due to its promising neuroprotective action demonstrated in *in vitro* and animal tests. Neurological diseases find their origin in neuroinflammation, a process that triggers the activation of microglial cells and astrocytes, leading to the release of pro-inflammatory cytokines that can initiate a cascade of signaling pathways. Nevertheless, phytocannabinoids have emerged for their notable anti-inflammatory activity, exerting their influence by inhibiting the release of glutamate at synapses. This mechanism is pivotal, as it prevents neuronal damage and thus the development of neurological conditions such as Alzheimer's Disease (AD), Parkinson's Disease (PD), Huntington's disease (HD) and Epilepsy. The neuroinflammatory process stems from various sources, including pro-inflammatory cytokines, pathogenic molecules like lipopolysaccharides (LPS), and the lipoprotein receptor-related protein (LRP-1). For instance, LPS acts through the Toll-Like Receptor 4 (TLR4), triggering microglial activation and subsequent synthesis of pro-inflammatory cytokines such as IL-1β, IL-4, TNF-α, NO, and ROS. This scenario manifests a cytotoxic effect, exacerbating the neuroinflammatory framework linked to central nervous system (CNS) disorders.^24^

Alzheimer's disease (AD) is characterized by the buildup of amyloid-β and hyperphosphorylation of tau proteins, as well as the presence of neuroinflammation and oxidative stress.^50^ The interaction of CBD with neurotransmitter systems such as glutamate receptors (NMDA receptors, 2-amino-3-(4-butyl-3-hydroxyisoxazol5-yl)propionic acid (AMPA) receptors, and the serotonergic receptor (5-HT1A) emphasizes its use in the treatment of Alzheimer's disease.^51^^,^^52^ It has been reported that CBD reduces the production of Aβ by activating peroxisome proliferator-activated receptor-γ (PPARγ) whereas Δ^9^-tetrahydrocannabinol (△^9^-THC) inhibits Aβ aggregation, yet with limited clinical evidence.^24^

A study carried out by Esposito et al. (2011)^53^ explored the effect of CBD against the peroxisome proliferator-activated receptor-c (PPARc) which may be involved in the etiology and characteristics of Alzheimer's disease. PPARs are part of the hormone receptor family and their typically regulated by steroids and lipid metabolites, being expressed at low levels in the central nervous system (CNS). Research shows that PPARc is higher in some pathological conditions including Alzheimer's disease. The use of CBD in an animal model showed that in addition to demonstrating stimulation of neurogenesis, this compound has the ability to provide protection through the activation of PPARc, which leads to a reduction in reactive glucose levels and, as a result, reduces neurodegeneration.

The endocannabinoid system (ECS) plays a critical role in several physiological processes, including memory, mood, appetite, and pain sensation. It is highly expressed in brain regions such as the hippocampus and cortex, which are critical for memory and learning. Interaction of cannabinoids with the ECS in the brain has been described to influence cognitive function. Recent findings in rodent models of AD show promising effects of cannabinoids in reducing the deposition of amyloid plaques. Besides, cannabinoids appear to stimulate neurogenesis in the hippocampus.^54^ Suliman et al. (2018)^55^ reported that different doses of Δ^9^-THC fostered neurogenesis in the hippocampus of rats, followed by the improvement of cognitive behaviour of animals observed in behavioural test and molecular perspective.

In a study published by Bosnjak Kuharic et al. (2021),^56^ analysing data from heterogeneous placebo-controlled trials, concluded that no effective outcomes about any beneficial or harmful effects can be drawn from the use of cannabinoids in dementia. Further clinical data need to be obtained from patients undertaking this kind of treatment over longer time to run a more robust methodological analysis. Timler et al. (2020)^57^ designed a randomised, double-blind crossover trial protocol for patients suffering from dementia, receiving a dose of either a cannabinoid-based oil (3:2 delta-9-tetrahydrocannabinol:cannabidiol) or placebo which was registered with the Australian New Zealand Clinical Trials Registry. The registration number is ACTRN12619000474156. Challenges still need to be addressed in particular the definition of the therapeutic window for the dosage and time of treatment, together with the understanding of the mechanism of action, potential side effects and long-term impact using this type of treatment.

Parkinson's Disease (PD) ranks as the second most prevalent neurodegenerative condition after AD. PD's typical symptoms include muscle rigidity, movement disorders, tremors, psychological manifestations, cognitive impairment, sleep disturbances, and somatoform autonomic dysfunction, among others.^58^ Models of PD have shown an increase in the expression of CB2 receptors and microglial activation, suggesting potential dysregulation in the cannabinoid system.^59^ Notably, patients with PD exhibit increased CB2 receptor expression, while CB1 receptors remain relatively stable. This observation suggests that CB2 receptors may play a more prominent role than CB1 receptors in regulating inflammation associated with PD.

**Fig. 2:**
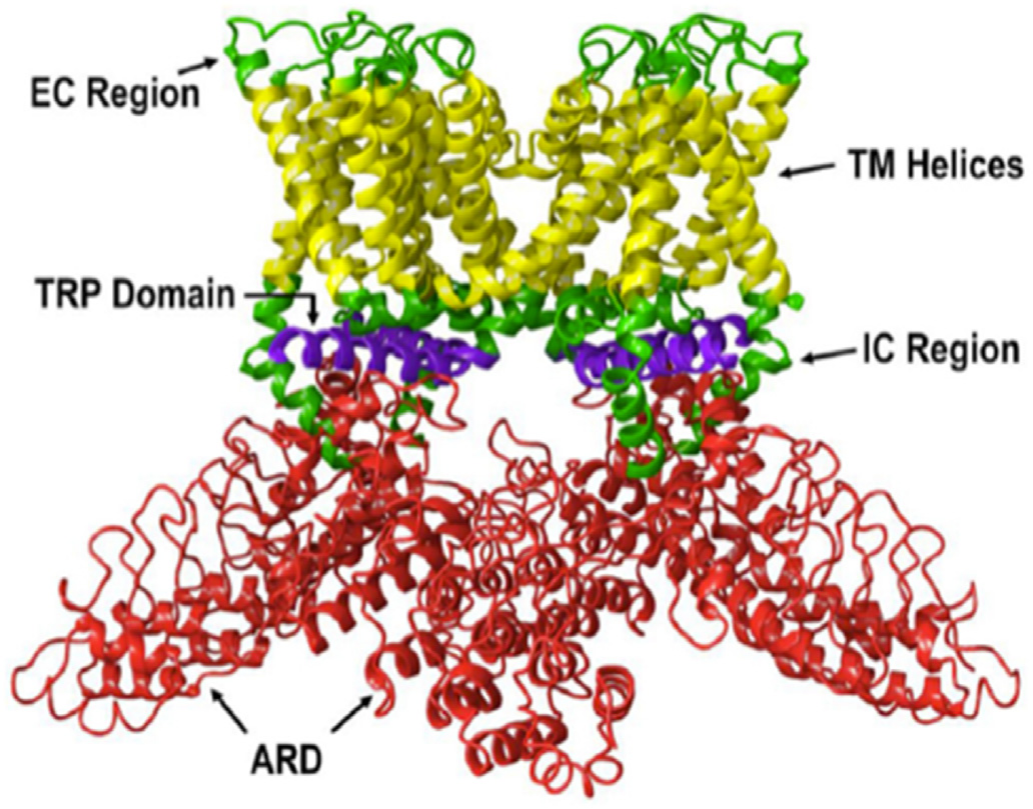
Representation of lipid of TRPV1 adapted from PDB: 3J5P. Ankyrin repeat domain (ARD) is shown in red, transmembrane helices are shown in yellow, the TRP domain is shown in purple, and intracellular regions (ICRs) and extracellular regions (ECRs) are shown in green.^64^

Regarding effects on Huntington's disease (HD), improvement of motor symptoms (e.g., dystonia) cannabinoids were reported to promote improvement of care, gait and fine motor skills and weight gain in patients.^60^ Cannabis also has a long history of anti-epileptic effects. Literature suggests that CBD acts as an agonist for Transient Receptor Potential Vanilloid 1 (TRPV1) channels, which are responsible for regulating Ca2+ channels.^61^ The use of CBD is also related to the treatment of seizures, promoting a reduction in the frequency and severity of these events.^47^ Studies report that the anticonvulsant activity of CBD may be related to the ability to reverse the dependent inhibition in the transient receptor potential (TRP) channels induced by pentylenetetrazole that develops antagonistic action of the transient receptor potential vanilloid 1 (TRPV1) responsible for neuroprotection (Fig. 2). Despite promising results, the anticonvulsant mechanism of action of CBD is still not fully elucidated.^46^^,^^62^^,^^63^

The anticonvulsant action of CBD was also evaluated in a study carried out by Kaplan et al., 2017^65^ using the Dravet model (SD) in mice. The results showed that CBD effectively reduced the duration and severity of seizures in the animals. The anticonvulsant mechanism was related to the participation of CBD in inhibitory neurotransmission occluded by a GPR55 antagonist, suggesting the participation of this lipid-activated G protein-coupled receptor responsible for the anticonvulsant action.

In addition to CNS-related effects, CBD also has antioxidant and anti-inflammatory properties. The antioxidant properties of CBD are regulated through ionic free radicals, which capture free radicals or transform them into less reactive forms, , directly and indirectly, regulating the redox state that interacts with other molecular targets associated with the redox system.^66^ A study developed by^67^ decided to evaluate the anti-inflammatory effect of phytocannabinoids, cannabidiol (CBD), and cannabigerol (CBG) alone and in combination through a bacterial lipopolysaccharide (LPS)-induced lung disease model in adult male Dunkin-Hartley guinea pigs. The actives CBD (10, 50, or 100 mg/kg), or CBG (10, 50, or 90 mg/kg) were administered intraperitoneally (IP) or orally (PO). The results showed that CBD and CBG had an anti-inflammatory effect on the animals' lungs, reducing the ability of LPS to induce neutrophil infiltration. Studies suggest that canonical cannabinoid receptors, CB1 and CB2 inhibit the activity of adenylate cyclase and thus decreasing the intracellular concentration of Ca2+ that controls the activation of several transcription factors thus regulating the expression of various cytokines such as IL-2, IL-4, and IFNγ, which affect cellular inflammatory responses.^66^^,^^68^^,^^69^

Another phytocannabinoid present in *Cannabis sativa* and widely explored for presenting several pharmacological properties is Δ9 -tetrahydrocannabinol (THC). THC was isolated in 1964 and over the years researchers have discovered its effects such as; hypolocomotion, hypothermia, catalepsy, and analgesia which are associated with CB1 receptor activation.^70^^,^^71^

The pharmacological effects of CBD and THC are also explored on the tumor cell inhibition front. Milian et al., 2020^72^ explored the effect of the CBD and THC combination on cells from patients with non-small cell lung cancer (NSCLC). This study aimed to evaluate the relationship between CB1 and CB2 expression and the effect of phytocannabinoids on proliferation, epithelial-to-mesenchymal transition (EMT), and *in vitro* migration in lung cancer cell lines (A549, H460, and H1792). According to the EGFR proliferation and expression test in lung cancer cells the concentrations of THC and CBD separately and in combination significantly inhibited the proliferation of A549 cells (dose-dependent), however only the combination of THC and CBD significantly decreased the EGFR expression in A549 and H460 cells. Given the results was possible to observe that CB1 and CB2 have the potential as biomarkers for the treatment of patients with NSCLC as well as the use of phytocannabinoids THC and CBD have the potential to suppress the proliferation of cancer cells.

The use of phytocannabinoids especially CBD and THC, has been extensively explored in recent decades, mainly for their potential effects that can be used in the treatment of various diseases that affect the CNS, in addition to their effects on cell proliferation, anti-inflammatory and/or analgesic. In this sense, the continuity of research that explores the pharmacological effects and that seek to elucidate a better understanding of their mechanisms of action and interaction with the receptors makes these phytocannabinoids important compounds for the development of new drugs.

### 3.1 Phytocannabinoids

Phytocannabinoids are compounds found mainly in the *Cannabis sativa* and *Cannabis indica* that have similar chemical structures but with specific therapeutic properties. In conjunction with the CB1 and CB2 receptors that are present in the endocannabinoid system, phytocannabinoids are widely manifested and differ in specific functions, localization, and signaling mechanisms.

One of the main effects is the anticonvulsant effect of cannabinoids, especially in CBD and THC.^73^ A seizure is a sudden change in the human brain that cannot be controlled, leading to symptoms such as confusion, fear, anxiety, muscle twitching, and unconsciousness. THC may decrease the effect of these seizures by being an agonist of the CB1 receptor and acting directly on the brain.^29^ In addition, phytocannabinoids can also help in the treatment of cancer, as they have anti-inflammatory and antioxidant activities, which are important functional properties for the inhibition of cell or tumor growth.

### 3.2 Endocannabinoid system (ECS)

Advances in studies involving the therapeutic use of *C. sativa* favored the discovery of the endogenous cannabinergic system, later called the endocannabinoid system. The endocannabinoid system is a lipid signaling system that has a regulatory function and influences the metabolism and physiology of several systems through anabolic actions that lead to protein and glycogen synthesis.

This system is composed of endocannabinoids (anandamide and 2-AG) derived from arachidonic acid (AA) and exocannabinoids (Δ-9-tetrahydrocannabinol, Δ-8-tetrahydrocannabinol, cannabidiol, and cannabinol) derived from the plant. Both systems are ligands for CB1 and CB2 receptors.^74^

This system (ECS) discovered in humans in the mid-1990s is composed of signaling molecules, proteins involved in their synthesis, catabolism, and transport, and receptors.^75^^,^^76^ The main functions of the ECS are related to the modulation of the nervous and immune systems, which are involved in several physiological processes, such as memory, sleep, appetite, learning, and hormonal release, that is, ranging from lipid metabolism and appetite to neuroprotection and neurogenesis.^77^

## 4 Therapeutic use

The therapeutic use of Cannabis is allowed in several countries around the world. The World Health Organization (WHO) indicates that several studies demonstrate the therapeutic effects of cannabinoids. According to research, there is substantial evidence on the use of Cannabis or cannabinoids in different health treatments. This compound has been shown to be effective in treating chronic pain in adults, reducing nausea and vomiting caused by chemotherapy, and improving symptoms of spasticity in patients with multiple sclerosis. There is also moderate evidence to suggest that it can improve short-term sleep outcomes in individuals with sleep disorders related to obstructive sleep apnea syndrome, fibromyalgia, chronic pain, and multiple sclerosis. In addition to the limited evidence for increasing appetite and decreasing weight loss associated with HIV/AIDS, improving symptoms of Tourette's syndrome and improving anxiety symptoms in individuals with social anxiety disorder.^78^^,^^79^

Despite the potential therapeutic benefits of Cannabis, research into its use is still limited due to restrictions on access to the quantity, quality, and type of Cannabis products needed to address specific health-related research questions. Another type of limitation found in different countries has to do with regulatory barriers. In the US, for example, there are limited sources for cannabis and cannabinoid-based study drugs, as well as limited resources to support studies. All these limitations make it difficult to carry out research aimed at answering the most pressing public health questions related to the therapeutic use of Cannabis and cannabinoids. It is important to note that the WHO made a series of recommendations to the United Nations to update the scope of control of Cannabis and related substances, reflecting the emerging therapeutic role of Cannabis-based medicines.^80^^,^^81^

## 5 Biomedical applications

**Fig. 3:**
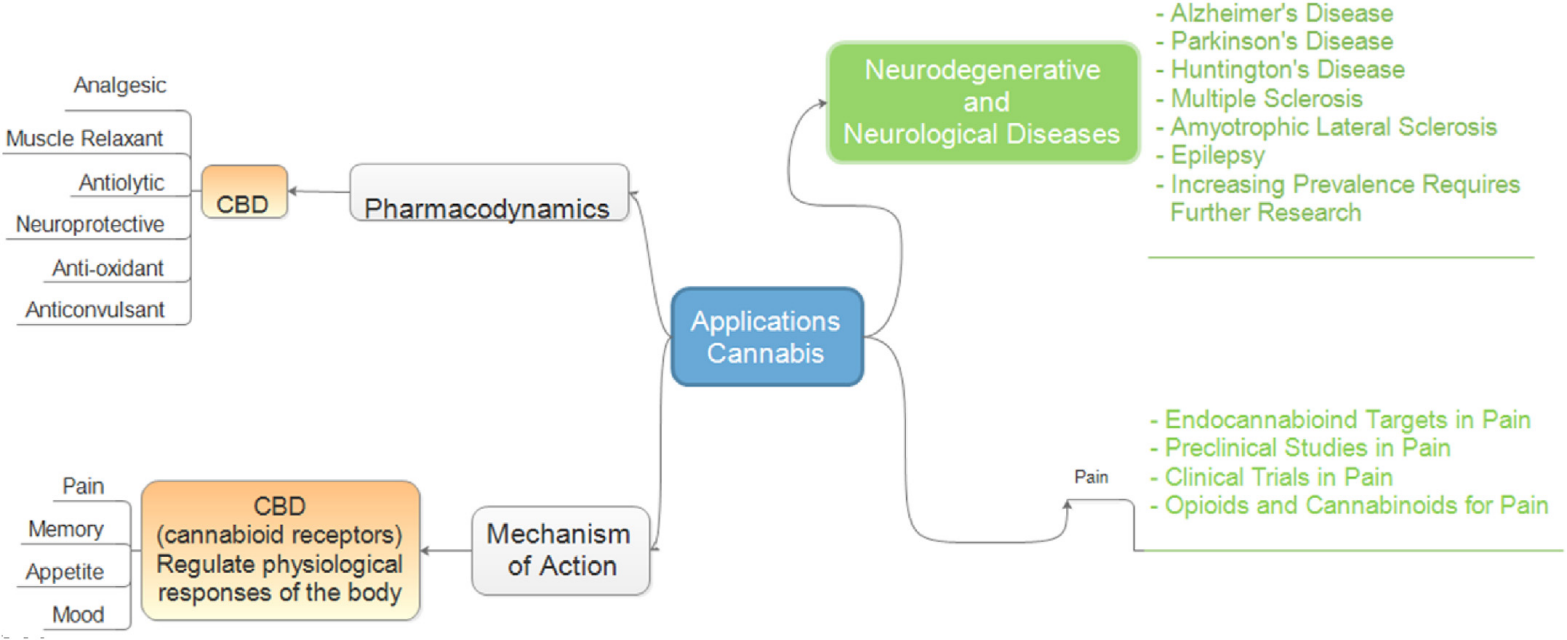
Biomedical applications of Cannabis derivatives.^88^

Cannabis (*sativa species L.*) has been used in different sectors, such as food, fiber, and especially in folk medicine due to its bioactive compounds.^12^^,^^82^, ^83^, ^84^, ^85^ These characteristics attracted cannabis use in the biomedical area, where numerous works have been developed to verify its potential.^29^^,^^86^^,^^87^ According to this, cannabis is currently studied for the treatment of several diseases (Fig. 3).

Bibliographic research conducted by Souza et al. (2021)^89^ verified the application of cannabis compounds in the therapeutic area: appetite suppressant for obesity/overweight treatment, treatment of nausea and vomiting in cancer and AIDS patients, treatment of insomnia, neuropathic pain, stress, spasms, multiple sclerosis, and depression, treatment of spasticity related to multiple sclerosis, and treatment of seizures associated with Lennox - Gastaut and/or Dravet syndrome.

The most active compounds present in Cannabis plants are cannabinoids and terpenes (CBD, Δ9-THC, cannabidiol acid - CBDA, cannabinol - CBN, and cannabigerol - CBG). Such substances have therapeutic potential, being used to treat various disorders (metabolic, neurodegenerative, movement), anorexia in HIV patients, nausea, and pain reliever for cancer patients.^90^

Cannabinoids extracted from *Cannabis sativa* L. have therapeutic effects, but their use is limited due to their psychotropic effects. However, research in targeted drug delivery systems has been an ally to minimize the undesirable psychotropic effects of some cannabis constituents. Currently, drugs containing cannabinoids (Δ9-THC and CBD) have been used for the treatment of various pathological disorders, such as cancer, neurodegenerative and dermatological diseases, and viral infections.^29^^,^^91^

Cannabinoid-based hydrogels containing *Cannabis sativa* L. extracts have their impact on keratinocytes evaluated by the content of biologically active compounds (e.g., phenols, flavonoids, chlorophylls, and cannabinoids). Such work found that the extracts helped prevent the degradation of collagen and elastin fibers, also presenting antioxidant properties.^92^

The biogenic synthesis of silver nanoparticles (AgNPs) is favored by the synergistic effect of terpenes, flavonoids, and cannabinoids from *Cannabis sativa L.*^93^^,^^94^ In this case, the AgNPs with cannabis extract showed antioxidant capacity, besides antibacterial activity against several human pathogens: *Escherichia coli*, *Klebsiella pneumoniae*, *Pseudomonas fluorescens*, and *Staphylococcus aureus*.

A study presented that cannabis extracts when properly prepared can have expressive *in vitro* bioactivity, significantly decreasing the viability of cancer cells (colon), and protecting healthy cells from cytotoxic effects.^95^^,^^96^

Collagen hydrogels loaded with AgNPs and *Cannabis sativa* oil extract presented anti-inflammatory and antioxidant activities, besides analgesic effects. These results affirmed the biocompatibility and antimicrobial activity of this nanocomposite, creating a promising biomaterial for wound healing and infection treatments.^97^

Phytocannabinoids also exhibit remarkable anti-inflammatory effects through CB2 inhibitory activity, with CBD, CBN, and Δ9-THC presenting antiviral effects against COVID-19, being applied as immunosuppressive and anti-inflammatory extracts for this specific respiratory treatment.^98^

The phytocannabinoids also show an important inflammatory activity via experimental models *in vitro* and *in vivo* in the treatment of several degenerative diseases related to inflammation. CBD has also been applied in the treatment of acne, its activity in reducing inflammation, modulating the immune system, and the manifestation of immune responses in human sebaceous glands has drawn the attention of several researchers.^99^^,^^100^ evaluated the antimicrobial, anti-inflammatory, and anti-lipogenic effects of hexane extracts from *Cannabis sativa* L. seeds against the Propionibacterium bacteria responsible for the inflammatory response that causes acne and lipogenesis. For the greenhouse, the seeds were dried and ground and later added in different volumes of hexane, after this process the extract was filtered and the solvent was rotary evaporated at 70 °C for 10 min. The anti-inflammatory effect of cannabis extract was evaluated through the expression of iNOS and COX-2 enzymes and pro-inflammatory cytokine IL-1β using the western blotting method with HaCat cells infected by *Propionibacterium acnes* (*P. acnes*, KCTC). The results showed that the cannabis seed extract exerted an anti-inflammatory activity through the regulation of enzymes related to the process in addition to the decrease in the expression of phosphorylated IKKα/β, IκBα, and NF-κB induced by *P. acnes*. The results also showed that the hexane extract of cannabis seeds reduced about 40% of MMP-9 activity compared to Hs68 cells treated with P. acne at 0.15% HSHE which means an important capacity of phytocannabinoids present in reducing damage to the extracellular matrix (ECM) mainly in the release of metalloproteinases (MMPs) responsible for the digestion of various components of the ECM, giving the cannabis extract a promising treatment for acne in a possible replacement of antibiotics and retinoids and their risk of adverse reactions.

**Fig. 4:**
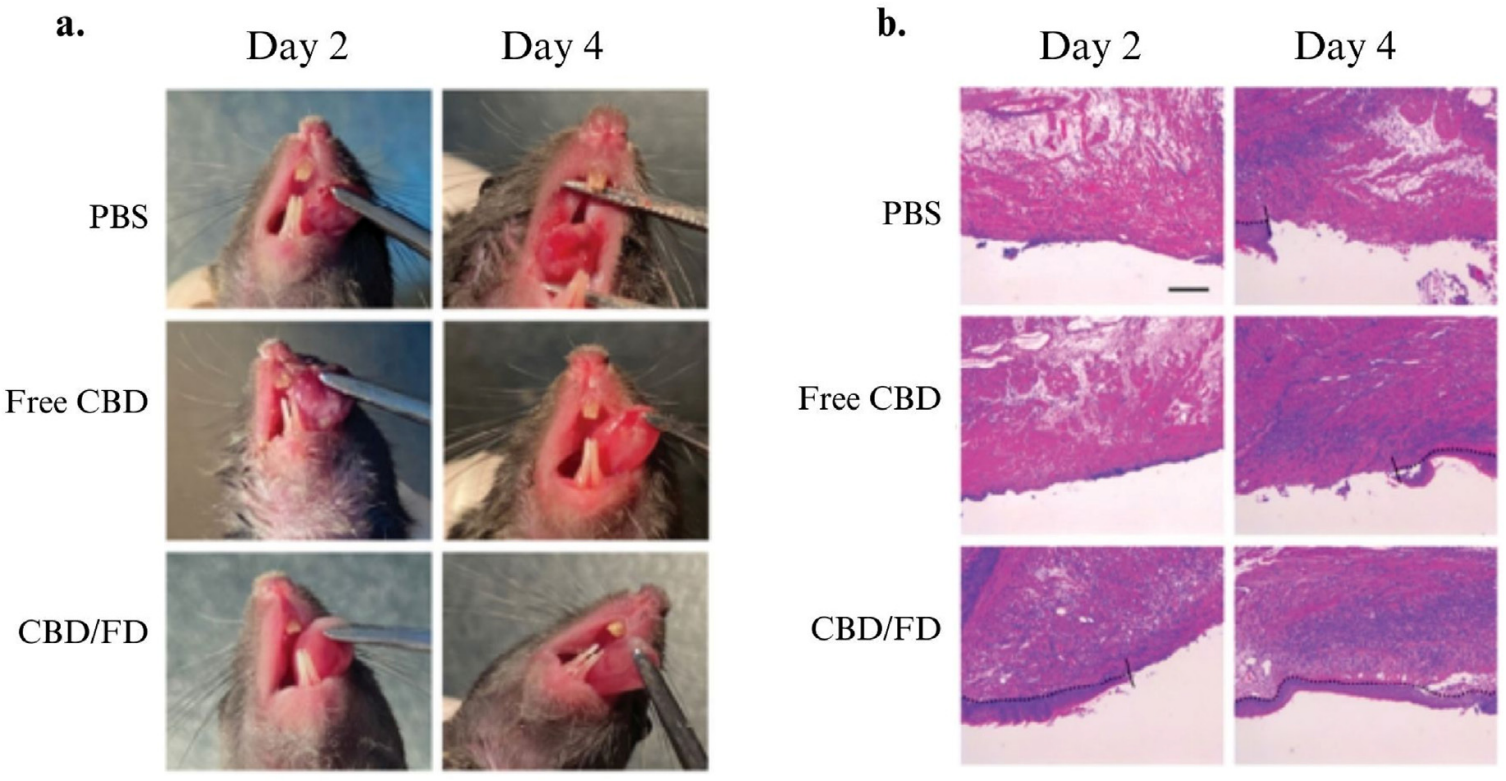
The therapeutic effect of CBD/FD nanomicelles on tongue ulcers after intravenous administration. (A) Drug administration and sampling. (B) Change in the ulcer area over time (n ¼ 5). Data are shown as mean ± SD. (C) Tongue ulcer images at 2 and 4 days post-administration. (D) Hematoxylin and eosin images of tongue ulcers. Solid lines indicate the ulcer boundary, and dotted lines indicate the epithelial-stromal boundary. Scale bar, 100 lm (modified with permission from ,^101^ copyright).

P-selectin expression, *in vitro* and *in vivo* assay were to verify the anti-inflammatory effects of CBD/FD (Fig. 4). Nanomicelles containing CBD showed a satisfactory encapsulation efficiency of around 88.8 ± 0.2% and a zeta potential of 24.45 ± 1.25 mV. The results of the assay that evaluated the *in vitro* anti-inflammatory and anti-inflammatory ability of FD micelles prescribed in this assay showed the important participation of P-selectin for the fucoidan-containing nanomicelles to reach inflammation, in addition to showing that Lipopolysaccharides (LPS) induced the enhancement of TNF-a and IL1b by HOK cells which had their levels reduced by CDB/FD nanomicelles. The effectiveness of CBD/FD nanomicelles in treating tongue ulcers in rats was assessed by monitoring the ulcer area on a daily basis following either intravenous injection or in situ application of free CBD or CBD/FD nanomicelles. The results showed that CBD/FD-containing nanomicelles led to a significantly lower degree of ulceration compared to PBS and free CBD controls, showing that CBD/FD-containing nanomicelles have a promising anti-inflammatory and wound-healing ability after being administered by drip or IV.

## 6 Marketed products

Pharmaceutical studies on cannabis increased in the 1990s thanks to the discovery of the endocannabinoid system (ECS), which is a set of networks that control physiological homeostasis. The two main receptors that belong to this system have the name cannabinoid receptor 1 (CB1) and cannabinoid 2 (CB2). The interest in this plant derives from the fact that since ancient times substances extracted from plants have been used for specific therapies and cannabis is one of these as it is rich in various phytochemicals that can help to treat or reduce pain in various diseases. The two receptors are the targets of the phytochemical components of cannabis, the best known, Δ9-tetrahydrocannabinol (Δ9-THC) and cannabidiol (CBD).^102^^,^^103^

These two receptors are normally found at the terminals of central and peripheral neurons, drugs composed of these phytochemical molecules that are extracted from the plant activate these receptors by targeting the orthosteric sites.^104^^,^^105^

In recent years, the clinical research concerned with the study of cannabinoids has increased significantly, leading to define these compounds as suitable for people who have neurological disorders, spasticity, and pain, nausea due to diseases such as cancer and arthritis.^106^^,^^107^

Pharmaceutical products, as shown in Table 3, contain phytocannabinoids below, contain phytocannabinoids,^107^ which are becoming popular as an alternative to traditional analgesics. CBD was discovered to have powerful anxiolytic and antidepressant properties. Furthermore, it has been shown that it increases neurogenesis (growth of brain cells) in key areas of the brain associated with anxiety and depression, Δ9-THC has antiemetic properties, is used as a post-traumatic treatment for stress-related disorders, and finally also as a stimulant for the appetite, Cannabidivarin as treatment of seizures, and also Cannabigerol as treatment of Huntington's disease, an inherited brain disease that affects the nervous system and causes emotional, cognitive and motor disorders.^102^^,^^107^

**Table 3:** Marketed pharmaceutical products derived from Cannabis.^108^

Drug	Manufacturer	Cannabis-Related Properties	Medical Use
**Mevatyl®**	Beaufour Ipsen Farmacêutica Ltda.	Oral spray with THC and CBD	Treatment spasticity to reduce pain in multiple sclerosis' patients
**Sativex®**	GW Pharmaceuticals	Oral spray with THC and CBD	Treatment of neuropathic pain and spasticity for multiple sclerosis' patients
			Analgesic effect for those with advanced cancer
**Dronabinol®/Marinol®**	Unimed Pharmaceuticals (Solvay Pharmaceutical)	Synthetic Δ9-THC	Reducing nausea and vomiting in cancer treatment
			Appetite stimulant
			Pain killer for neuropathic disease
**Nabilone®/Cesamet®**	Valeant Pharmaceuticals International	Synthetic cannabinoid	Reducing nausea and vomiting for patients in cancer treatment
**Dexanabinol®**	Solvay Pharmaceuticals (Abbott Laboratories in 2010)	Synthetic non-psychotropic cannabinoid	Brain neuroprotective agent
			Memory treatment for traumatic brain injury
**CT-3 (ajulemic acid)**	Indevus Pharmaceuticals	Synthetic cannabinoid	Treatment of spasticity and neuropathic pain
			Anti-inflammatory properties for chronic pain
**Cannabinol (formerly PRS-211,375)**	Pharmos	Synthetic cannabinoid	Anti-inflammatory for chronic pain
			Bladder control
**HU 331**	Cayman Chemical	Synthetic cannabinoid	Memory treatment
			Neurodegeneration treatment
			Appetite stimulant
			Reducing tumor pain
**Rimonabant®/Acomplia®**	Sanofi-Aventis	Synthetic cannabinoid	Anti-obesity (appetite reducer)
**Taranabant®**	Merck	Targets receptors in the brain linked to appetite	Anti-obesity (appetite reducer)

## 7 Cannabis prospects

### 7.1 Worldwide

After the legalization of Cannabis edibles, companies in the most diverse sectors have been analyzing the possibility of selling Cannabis-infused food products. Through research carried out by the official Cannabis use database, known as CANNES, it is possible to obtain data containing information about the use of the plant in 41 countries around the world. They report about 2,330 items of *Cannabis sativa* for medicinal use (75.41%), recreational use (8.35%), and use in food and beverages (7.29%).^83^

The demand for Cannabis-derived products has increased, with technological innovation and involvement of utmost importance to producers since it can help to provide possibilities for manufacturing vaping oils, gummies, edibles, and capsules, in addition, to assisting extraction techniques for pharmaceutical products.^109^

In 2022, the global Cannabis market was valued at $27.7 billion and is projected to grow to $82.3 billion by 2027, with a compound annual growth rate (CAGR) of 2.3% in terms of value. The medical and recreational cannabis markets are currently at the forefront of the global legalization movement. Considering the importance and global demand for the product, expanding medical uses, increasing research and development (R&D), and technological innovation. At the same time, it is expected to grow the social acceptance of Cannabis, while new technologies are expected to boost the global Cannabis market during the forecast period.^109^

As previously mentioned, CBD has been indicated in the treatment of various diseases. Furthermore, several applications involving the field of cosmetics and pharmaceuticals have been implemented, products such as creams, lotions, lubricants, and others are infused with cannabidiol to decrease muscle pain, joint pain, inflammation, headache, and cramping.^110^

The market for pharmaceutical and cosmetic products with therapeutic potential in North America and Europe has been expanding exponentially, due to the greater number of applications and benefits. Therefore, it is necessary to prove tolerability, safety, toxicity, efficacy, ideal dosages, and ideal delivery systems related to *C. sativa* pharmacological products through rigorous scientific studies.^111^ The trade of Cannabis-based medicines and products has significant economic potential, as it can generate substantial revenue and create jobs. Additionally, Cannabis has the potential to become a valuable commodity in Brazil.

### 7.2 Brazil

The Cannabis product market in Brazil is in the process of development and has been very promising and with high growth potential. For patients who are treated with plant-based medicines through medical prescription, there are three ways of obtaining them: importing, through associations, or pharmacies.

The trade-in cannabinoid-containing drugs in Brazil started in 2018 when ANVISA released the registration of the Mevatyl drug, an oral spray solution that is indicated for the treatment of symptomatic spasticity (moderate to severe) related to multiple sclerosis. This is manufactured by GW Pharma Limited, a UK company, but in Brazil, the company that has the registration is Beaufour Ipsen Farmacêutica Ltda., located in São Paulo.^112^

In 2019, ANVISA established a health authorization (RDC no 327/2019) for the manufacture and import of products based on Cannabis compounds for medicinal purposes to provide safe and quality products to the Brazilian population. This authorization also establishes requirements for the commercialization, prescription, dispensing, monitoring, and inspection of these products. Through this authorization, the product can be prescribed, but it has some requirements that may be for palliative care only. Its prescription is restricted to professionals doctors legally qualified by the Federal Council of Medicine (CFM).^113^

Most drugs sold in Brazilian pharmacies are imported, therefore, they are not easily accessible to everyone, as they have a high cost. The only product authorized by ANVISA, produced and marketed in the country, is Cannabidiol Prati-Donaduzzi. The phytocannabinoid present in the drug is exclusively CBD and its sale is subject to the presentation of a controlled numbering prescription. The handling of the product is carried out by the pharmaceutical industry, using highly purified raw materials and carrying out all the necessary quality tests, ensuring the safety of the drug.^114^

After some favorable court decisions, the Cannabis market in Brazil has been moving around R$130 million per year. Unlike countries that already legalized Cannabis, to have access to Cannabis in Brazil, preventive *habeas corpus* is required, which allows the consumption and cultivation of the plant to treat various diseases.^115^ Furthermore, the company estimates that in 4 years after the medical, industrial, and recreational regulation, the Cannabis sector would generate R$26.1 billion to the country's economy and contribute more than 328,000 formal and informal jobs.^115^

The market continues to heat up, with almost 100 patent applications in Brazil related to Cannabis and its phytocannabinoids, with 5% of them sent for the approval of ANVISA. The next step towards a complete market is the regulation of the plant's cultivation, which is already underway in Brazil through the PEC 399/2015, with the potential to become a great high-end commodity of economic and social importance for the country.

The search run on the Scopus database (5^th^ April 2023) resulted in 972 manuscripts published since then. Limiting the works to those affiliated with Brazilian institutions, the list was downsized to 396 manuscripts indexed on Scopus, out of which 30 were published already in 2023.

**Fig. 5:**
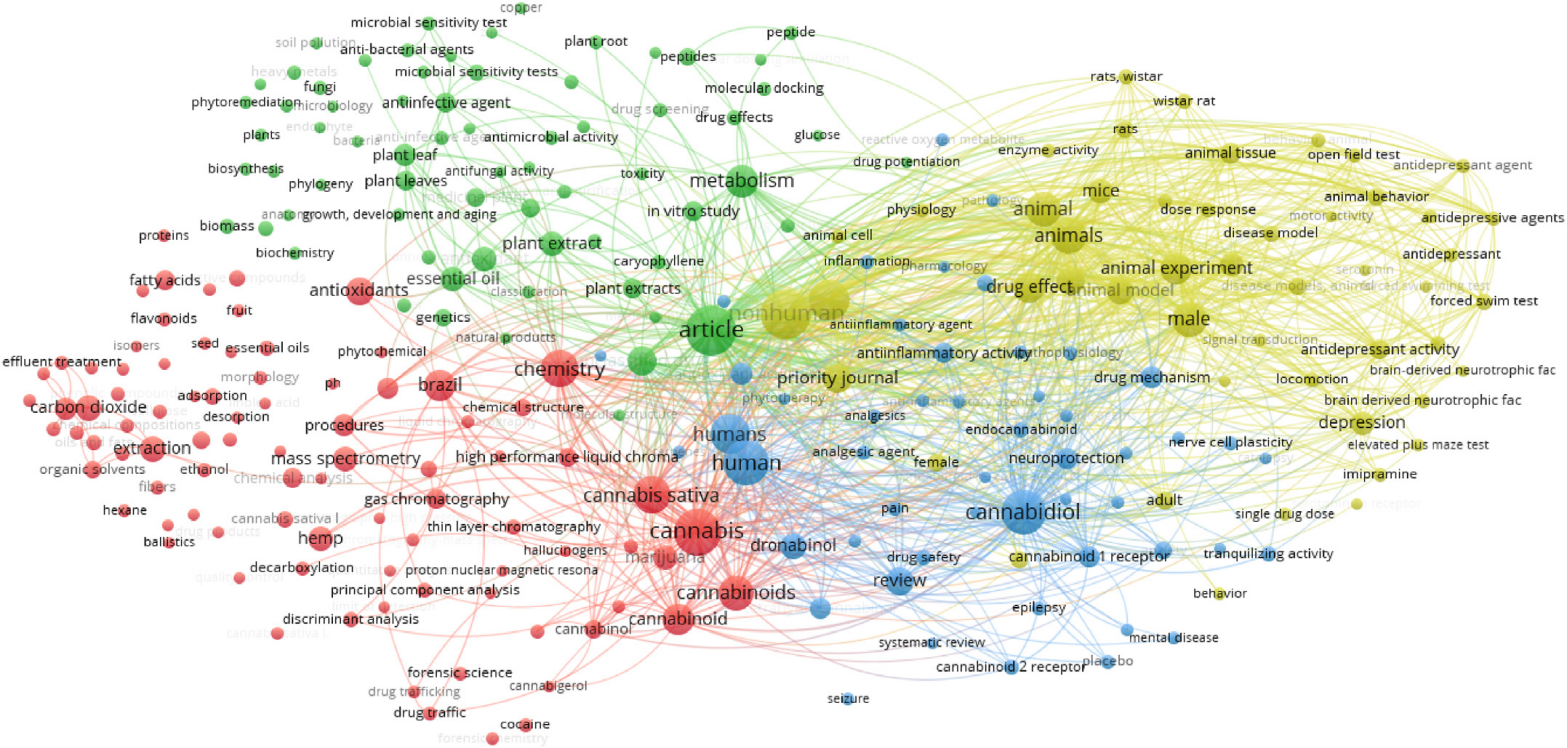
Bibliometric map obtained by VOSviewer software version 1.6.16 (https://www.vosviewer.com),^117^ using “*Cannabis Sativa* L.” and “Brazil” as searched terms, analyzing the works affiliated to Brazilian institutions, registered from Scopus database (5^th^ April 2023).

Fig. 5 shows the bibliometric map generated by the VOSviewer software,^116^^,^^117^ using the combination of the terms “*Cannabis Sativa* L.” AND “Brazil” as keywords of the analyzed 396 papers.

From the outputs shown in Fig. 5 (terms were searched in articles’ abstract and keywords), 259 co-occurrences were recorded, clustered into four categories namely, 89 items for the red cluster (e.g., cannabinoids, chemical composition, chemical analysis, cocaine, cultivation, drug traffic), 69 items for the green cluster (e.g., metabolism, *in vitro* study, drug effects, drug screening, plant extract), 51 items for the blue cluster (e.g., anti-inflammatory activity, anti-oxidant activity, anxiety, neuroprotective agents), and 40 items for the yellow cluster (e.g., *in vivo* study, animal experiment, behavior, brain-derived neurotrophic factor, depression).

### 7.3 South Africa

As the worldwide market of cannabis and hemp is thriving as the result of approved legislation and regulatory use, its prevalence and consumption in sub-Saharan Africa have recently become the object of study.^118^ However, African countries overall provide a significant contribution to the supply chain. According to the African Cannabis Report,^119^ the global market of cannabis and its related products is expected to reach $102 billion by 2026. Fortune Business Insights estimates a total of $197.74 billion by 2028.^110^ Among sub-Saharan countries, Lesotho is reported to be leading in medical cannabis, whereas the South African Health Products Regulatory Authority already issued 76 licenses for crop cultivation of medicinal cannabis.^120^ In 2021, the cannabis industry in the country had an estimated worth of R87.7 million, and it is expected to reach R406.3 million by 2026, a 5-years projection above 28%.^120^ These numbers are expected to stimulate the job market as well.

A study published in 2018 discussed the management of pain related to HIV-associated sensory neuropathy (HIV-SN) as one of the priority areas for the use of cannabinoids in South Africa.^121^ The selection of this condition was based on the high burden of HIV and HIV-SN in the country and the lack of available pharmacotherapy for neuropathic pain.

Given its favorable climate, South Africa is an attractive and potential player in global market production. However, it still needs to overcome regulatory and legal constraints to allow the use of cannabis and hemp in medicinal treatments. Relevant concerns are being raised in the country regarding its use in adolescents and pregnant women following liberalization.^122^

**Fig. 6:**
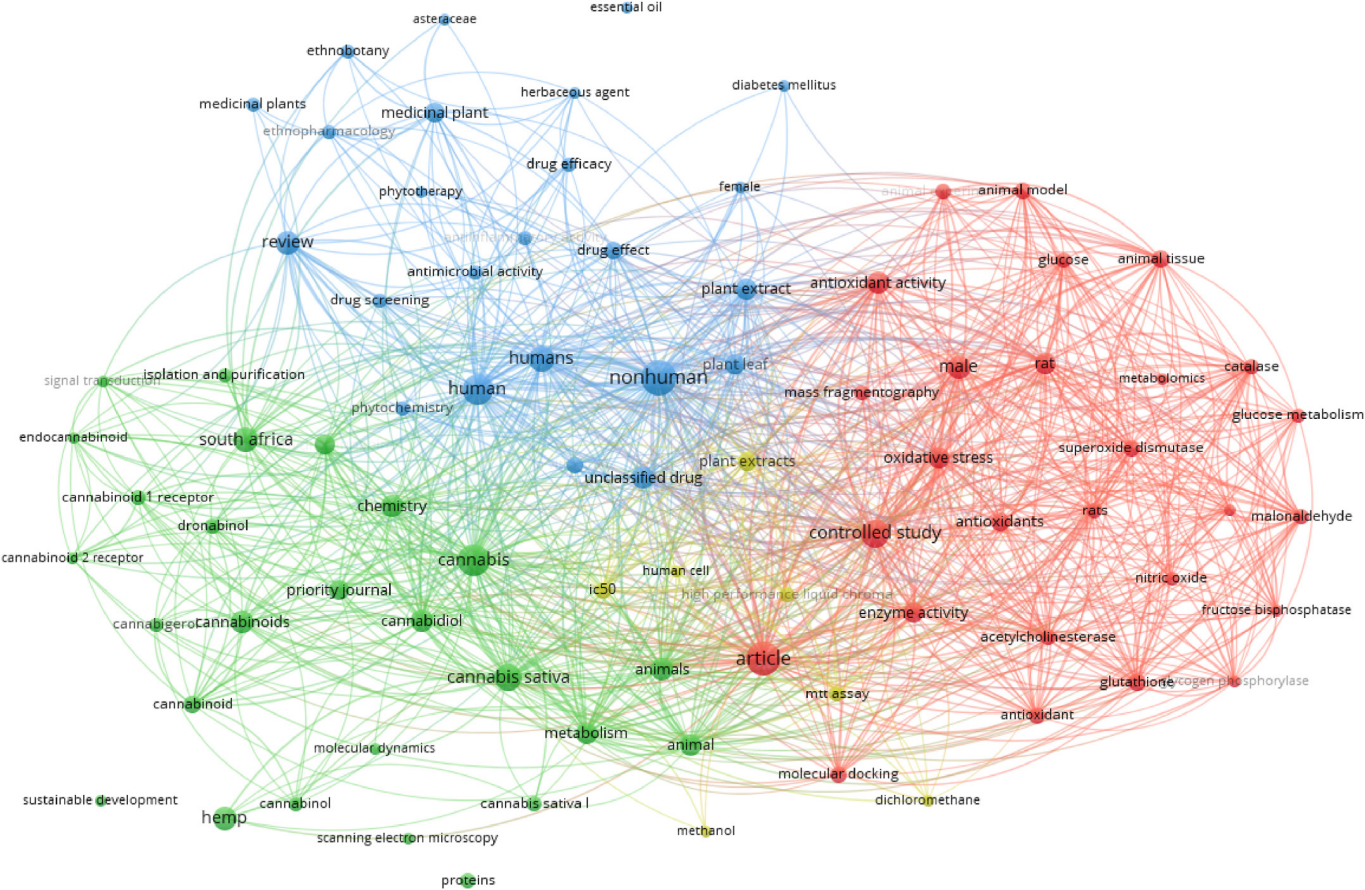
Bibliometric map obtained by VOSviewer software version 1.6.16 (https://www.vosviewer.com),^117^ using “*Cannabis Sativa* L.” and “South Africa” as searched terms, analyzing the works affiliated to South African institutions, registered from Scopus database (5^th^ April 2023).

Besides, from the safety point of view, cannabis products should be obtained free from organic solvent residues. A study published in 2022 analyzed the solvent residue contaminants of South African cannabis-based product samples ranging from crude extract, product development samples, and market-ready final products.^123^ The authors reported a significant concentration beyond the limits acceptable by the US Pharmacopoeia highlighting the need for product quality control. This study concludes the urgent need of educating the South African population about the risks associated with cannabis-based products.^123^ Fig. 6 shows the bibliometric map generated by the VOSviewer software,^116^^,^^117^ using the combination of the terms “*Cannabis Sativa* L.” AND “South Africa” as keywords resulting in 142 papers affiliated to South African institutions.

From the outputs shown in Fig. 6 (terms were searched in articles’ abstract and keywords), 84 co-occurrences were recorded, clustered into three categories namely, 27 items for the red cluster (e.g., animal experiment, animal model, controlled study), 26 items for the green cluster (e.g., cannabidiol, cannabinoids, dronabinol, metabolism) and 24 items for the blue cluster (e.g., anti-inflammatory activity, anti-microbial activity, drug effect, ethnopharmacology).

### 7.4 Canada

The Canadian government legalized medical marijuana access in 1999 via Section 56 exemption. The Marihuana Medical Access Regulations (MMAR) in 2001 allowed patients with severe illnesses to possess and produce medical cannabis. The Marihuana for Medical Purposes Regulations (MMPR) in 2013 established licensed producers to ensure quality-controlled cannabis. The Access to Cannabis for Medical Purposes Regulations (ACMPR) coexisted, enabling personal cultivation and licensed seller access. The Cannabis Act (Bill C-45) of 2018 legalized recreational cannabis, with medical patients using both systems. The country's emphasis on research, patient access, and product quality has positioned it as a leader in the global medical cannabis market. According to the authors, by the end of September 2019, 369,614 Canadians were registered with a licensed seller under the medical cannabis regulations, and an additional 29,193 Canadians were registered through Health Canada, allowing them to cultivate their own medical cannabis. In summary, a large number of Canadians have access to medical cannabis through licensed sellers or personal cultivation. Ongoing studies and clinical trials contribute to an expanding understanding of the medical potential of cannabis-derived compounds.^124^

### 7.5 United States of America

Industrial hemp (*Cannabis sativa* L.) cultivation is gaining significant attention due to its potential as an environmentally friendly and economically viable crop. Distinguished from marijuana by its low tetrahydrocannabinol (THC) content of less than 0.3%, hemp has seen a resurgence in the US following legislative changes that removed it from the list of controlled substances. As a result, hemp cultivation is now legal in 46 states and over 47 countries worldwide. This resurgence is driven by its versatility across industries and its potential to enhance soil health and contribute to sustainable agricultural practices.^125^

The renewed interest in hemp stems from its multifaceted benefits and adaptability. Beyond its varied uses, hemp has demonstrated the ability to rejuvenate the soil, suppress weeds, and even function as a natural pest repellent, aligning with organic farming practices. The 2018 US Farm Bill played a pivotal role in redefining hemp's status, fueling its widespread cultivation. With its global market predicted to double, hemp holds promise in industries like textiles, biofuels, and pharmaceuticals.^126^

### 7.6 Europe

A study in Canada and the USA found that CBD users are more likely to view CBD as beneficial for their health compared to non-users. While CBD is generally considered safe in Europe, there is limited research on the topic. In Germany, the only European country with available data on national CBD use, 4.3% of individuals have used CBD. Studies conducted in non-representative samples of populations have reported a prevalence of CBD use ranging from 10.9% to 14% in Europe, 26.1% in the US, and 16.2% in Canada.^127^

However, there's a notable scarcity of comprehensive data regarding the pricing of cannabis products within the European context. As global cannabis policies progressively shift towards legalizing both medical and recreational use, the need for accurate and well-structured data on potency and pricing becomes crucial. In response to this demand, the European Monitoring Centre for Drugs and Drug Addiction (EMCDDA) meticulously compiles annual datasets on cannabis potency and pricing across diverse European nations. This effort provides invaluable insights into the dynamic evolution of the cannabis market. The present study aims to quantitatively analyze changes in the potency, pricing, and value of cannabis resin and herbal cannabis across Europe from 2006 to 2016. By doing so, it significantly contributes to an improved understanding of the intricate interplay between cannabis market dynamics and the evolving landscape of policy paradigms.^128^

## 8 Conclusions

Cannabis is known worldwide for its medicinal applications. Several studies show an improvement in the effects and reduction of seizure episodes, pain reduction in patients with AIDS and cancer, anxiolytic, anti-nausea, and antioxidant activity. However, there are still a few products on the market containing phytocannabinoids, CBD, and THC, as well a few countries have legislation on their use and legalization in medicines and cosmetics. For example, in Brazil, it was only in 2019, that its medicinal use was approved, being only a drug containing CBD approved by local authorities, and that this product is not produced in Brazil. Legislation would be needed to support the medicinal use of Cannabis, as well as, there is a need to establish new and adequate regulation of Cannabis cultivation, which could generate many jobs and potential benefits for patients. Furthermore, CBD and THC products could constitute a potentially suitable approach for cancer therapy.

## Declaration of competing interest

The authors declare that they have no known competing financial interests or personal relationships that could have appeared to influence the work reported in this paper.
